# Glycaemic control and its associated factors in patients with type 2 diabetes in the Middle East and North Africa: An updated systematic review and meta‐analysis

**DOI:** 10.1111/jan.15255

**Published:** 2022-05-27

**Authors:** Odai Hamed Al‐ma'aitah, Daniel Demant, Samantha Jakimowicz, Lin Perry

**Affiliations:** ^1^ School of Nursing and Midwifery, Faculty of Health University of Technology Sydney Ultimo New South Wales Australia; ^2^ School of Public Health, Faculty of Health University of Technology Sydney Ultimo New South Wales Australia; ^3^ School of Public Health and Social Work, Faculty of Health Queensland University of Technology Brisbane QLD Australia; ^4^ Prince of Wales Hospital, South Eastern Sydney Local Health District Sydney Australia

**Keywords:** glycaemic control, Middle East, systematic review, meta‐analysis, type 2 diabetes mellitus

## Abstract

**Aims:**

To examine the patient‐related factors that have been linked to glycaemic control in people living with type 2 diabetes mellitus in Middle Eastern countries.

**Design:**

A systematic review and meta‐analysis.

**Data Sources:**

A computerized search was conducted using the databases MEDLINE (via PubMed and Ovid), EMBASE, Scopus and CINAHL to identify peer‐reviewed articles published in English between 1 January 2010 and 21 May 2020. On 28 June 2021, the search was updated with the same keywords and databases; however, no further relevant studies were identified.

**Review Methods:**

Extracted data were analysed using Review Manager 5.4.

**Results:**

The final sample consisted of 54 articles with a total of 41,079 participants. Pooled data showed an increased risk of inadequate glycaemic control in smokers [OR = 1.26, 95% confidence interval (CI): 1.05, 1.52; *p* = .010], obese patients (OR = 1.30, 95% CI: 1.10, 1.54; *p* = .002), patients with elevated waist to hip ratio (OR = 1.62, 95% CI: 1.16, 2.26; *p* = .004) and longer disease duration (OR = 2.01, 95% CI: 1.64, 2.48; *p* < .001). A lower risk of inadequate control was associated with physical activity (OR = 0.40, 95% CI: 0.24, 0.67; *p* < .001) and self‐management (OR = 0.49, 95% CI: 0.29, 0.82; *p* = .006).

**Conclusion:**

These findings highlight the opportunity to address factors to improve glycaemic control. Further longitudinal studies are required to better understand these variations, to assess all predictors of glycaemic control in participants with type 2 diabetes, and to provide a strong basis for future measures to optimize glycaemic control.

**Impact**

**What problem did the study address?**

Rising rates of diabetes, particularly in Middle Eastern regions, make it imperative to identify factors linked to glycaemic control among patients with Type 2 Diabetes in these areas.

**What were the main findings?**

Smoking, obesity, disease duration, physical activity and self‐management were the main factors linked to glycaemic control

**Where and on whom will the research have impact?**

Results will be relevant for policymakers, physicians, medical staff and patients with diabetes in the MENA region, informing them of key factors that should be considered concerning the management of diabetes.

## 1 INTRODUCTION

Diabetes is a major public health issue with an increasing burden globally (Johar et al., 2019). The latest data show that in 2019, 463 million adults were living with diabetes. It has been estimated that, without action to reduce the rising prevalence, by 2030, 578 million people will live with diabetes (Williams et al., 2019). One of the most affected areas is the Middle Eastern and North African (MENA) region, where approximately 54.8 million adults aged 20–79 were living with diabetes in 2019 (Williams et al., 2019). It was estimated that the number of patients with diabetes in the MENA region will reach 76 million by 2030 (Williams et al., 2019). Effective management of diabetes is, therefore, a priority for the countries of this region.

If not properly controlled, diabetes and its complications can result in frequent hospital admissions and premature death. Glycaemic control is a fundamental determinant of the prognosis of diabetes (Mamo et al., 2019). As a result, it is targeted by physicians to prevent diabetes‐related complications and mortality (Khattab et al., 2010). A wide variety of factors are associated with glycaemic control among participants with type 2 diabetes mellitus (T2DM); these include demographic factors such as age, gender, marital status and education (Abdullah et al., 2020; Aghili et al., 2016). Lifestyle parameters have also been linked with T2DM, such as smoking, obesity and physical inactivity (Al Slamah et al., 2020). In addition, correlations have also been noted between psychological status (e.g. depression and anxiety) and glycaemic status (Ahmadian et al., 2018; Al Hayek et al., 2014). Many of these factors have the potential to be influenced by the local context and cultural considerations. However, little information is available to inform priorities for the MENA region and clinical guidelines to direct healthcare professionals on how this information may be used to improve health and prevent or delay the onset of the complications of diabetes.

## 2 THE REVIEW

### 2.1 Aims

Given the high and rising prevalence of T2DM in the MENA region, and to minimize cultural and geographic heterogeneity, this systematic review and meta‐analysis was conducted to examine the patient‐related factors that have been linked to glycaemic control in people living with T2DM in the MENA region, to make recommendations for locally appropriate diabetes care.

### 2.2 Design

This systematic review and meta‐analysis followed the Preferred Reporting Items of Systematic Reviews and Meta‐analysis (Moher et al., 2016). Review methods and analyses were conducted in accordance with international recommendations (Agency for Healthcare Research and Quality, 2014; Higgins et al., 2019). The protocol of this study has been registered in PROSPERO (https://www.crd.york.ac.uk/prospero/; ID:CRD42021227250).

### 2.3 Search methods

A search strategy was developed based on the format of PEO: P (population), E (exposure, in this review interpreted as characteristics) and O (outcome). The search strategy was structured as follows:

P: Participants with T2DM living in Middle Eastern countries [Jordan, the United Arab of Emirates, Bahrain, Iraq, Saudi Arabia, Kuwait, Yemen, Iran, Syria, Israel, Oman, Palestine, Qatar, Lebanon, Egypt and Turkey (TeachMideast, 2020)].

E: Exposure (or characteristics) included demographic details (age, gender, race or ethnicity, education, marital and employment status); clinical characteristics (insulin use, duration of diabetes, body mass index and waist‐hip ratio); behavioural variables (smoking, diet, and physical activity); and mental and physical wellbeing characteristics such as anxiety and depression. Characteristics were chosen where they were examined in included studies, where there was a recognized rationale for a potential link to glycaemic control.

O: Outcomes sought were glycaemic control.

A computerized search was conducted using databases MEDLINE (via PubMed and Ovid), EMBASE, Scopus and CINAHL identify peer‐reviewed articles published in English between 1 January 2010 and 21 May 2020. On 28 June 2021, the search was updated with the same search strategy and databases; however, no further relevant studies were identified. Addtionally, all reference lists of included and any review articles were checked for potentially relevant studies. MESH terms were applied, and synonyms identified and used as keywords, linked with Boolian operands, as follows:

LINE 1: (“Non‐insulin‐dependent diabetes”, “Diabetes Mellitus, Type 2”, “Type II diabetes”, “Type 2 diabetes”, “NIDDM”).

AND

LINE 2: (“Middle East” OR “Jordan” OR “UAE” OR “Bahrain” OR “Iraq” OR “Saudi Arabia” OR “Kuwait” OR “Yemen” OR “Iran” OR “Syria” OR “Israel” OR “Oman” OR “Palestine” OR “Qatar” OR “Lebanon” OR “Egypt” OR “Turkey”).

AND

LINE 3: (“Glycaemic control”, “Glycemic Control”, “Blood Glucose”, “Haemoglobin A1c”, “HBA1c”).

NOT

LINE 4: (child* or kid* or adolescen*).

### 2.4 Eligibility criteria

To be included in the review, studies had to meet the criteria set out in the PEO framework. Studies were also required to be observational in design (case–control, cohort or cross‐sectional studies). Studies that were not available in English; animal studies; case‐reports or not full reports of primary research (e.g. conference abstracts and review articles) were excluded.

### 2.5 Search outcomes

Citations were downloaded into EndNote x9, and duplicate studies were removed using the “remove duplication” function. Two independent reviewers (O. H. A. and S. J.) screened the retrieved articles in two steps. First, only the titles and abstracts were independently screened to remove papers not meeting eligibility criteria. Then, the full‐texts of the remaining papers were retrieved for full‐text screening of their eligibility for inclusion. Full‐text screening focused predominantly on the methods and results sections to confirm the eligibility of the study and provision of relevant data. Any discrepancies between reviewers' decisions were addressed in discussion of the eligibility criteria with the third reviewer (L. P.).

### 2.6 Data abstraction

A standardized extraction form was prepared using MS excel (Microsoft), including the domains shown in Table 1. Data extraction was done independently by two investigators (O. H. A. and S. J.), with any discrepancies resolved in discussion with a third reviewer (L. P.). Missing odds ratios were calculated with review manager 5.3 using the required data (event and total of experiment and control groups). Missing standard deviations (SD) were calculated from the standard error (SE) with the following formula:

SD=SE×√N,

and from the confidence interval (CI) using this formula:

$$\mathrm{SD}=\surd N\times \left(\text{upper limit}-\text{lower limit}\right)/3.92.$$

**TABLE 1:** Summary of included studies and patients

Cross‐sectional studies								
Study ID	Selection				Comparability	Outcome		Quality score
	Ascertainment of the exposure (risk factor)	Non‐respondents	Sample size	Representativeness of the sample	The subjects in different outcome groups are comparable, based on the study design or analysis. Confounding factors are controlled	Assessment of the outcome	Statistical test	
Abdullah et al. (2020)	**	*	*	*	**	**	*	10
Abuhegazy (2017)	**			*	**	**	*	8
Adham et al. (2010)	**	*	*	*	**	**	*	10
Aghili et al. (2016)	**	*		*	**	**	*	9
Ahmadian et al. (2018)	**				**	**	*	6
Al‐Hayek et al. (2012)	**			*	**	**	*	8
Al Saweer (2015)	**			*	**	**	*	8
Al Slamah et al. (2020)	**	*	*	*	**	*	*	10
ALaboudi et al. (2016)	**	*		*	*	**	*	8
Al Balushi et al. (2014)	**	*		*	**	**	*	9
Albasheer et al. (2018)	**	*	*	*	*	**	*	9
Al Dossari et al. (2019)	**	*	*	*	*	**	*	9
AL‐Eitan et al. (2016)	**			*	*	**	*	8
Al Hayek et al. (2014)	**			*	**	**	*	8
Ali & Shahwan (2013)	**				**	**	*	7
Al‐Lawati et al. (2012)	**		*	*	*	**	*	8
Almetwazi et al. (2019)	**			*	*	**	*	7
Al‐Mukhtar et al. (2012)	**			*	*	**	*	7
Awwad Al qahtani et al. (2015)	**			*	*	**	*	7
Alqudah et al. (2019)	**		*	*	*	**	*	8
Alramadan et al. (2018)	**		*	*	*	**	*	8
Al‐Rasheedi (2014)	**		*	*	**	**	*	9
Alromaihi et al. (2019)	**			*	*	**	*	7
Al‐Shahrani et al. (2012)	**			*	*	*	*	6
Azadi et al. (2020)	**	*	*	*	*	**	*	9
Baltaci et al. (2012)	**			*	**	**	*	8
Baltaci et al. (2015)	**			*	**	**	*	8
Channanath (2018)	**			*	*	**	*	7
Cosansu & Erdogan (2014)	**			*	**	**	*	8
Greenberger et al. (2014)	**				*	**	*	6
Gucuk (2016)	**			*	**	**	*	8
Habib (2013a,b)	**			*	*	**	*	7
Jahanlou & Karami (2011)	**			*	*	**	*	7
Khattab et al. (2010)	**			*	*	**	*	7
Maddah & Attarpour (2016)	**			*	*	**	*	7
Ibrahim et al. (2020)	**			*	*	**	*	7
Mirahmadizadeh et al. (2020)	**	*	*	*	**	**	*	10
Mosleh et al. (2017)	**		*	*	**	**	*	9
Nemeh et al. (2011)	**		*	*	**	**	*	9
Noureddine et al. (2014)	**			*	**	**	*	8
Al Nozha (2014)	**		*	*	**	**	*	9
Qteishat & Ghananim (2016)	**			*	**	**	*	8
Radwan et al. (2018)	**		*	*	**	**	*	9
Saad et al. (2017)	**			*	**	**	*	8
Saghir et al. (2019)	**		*	*	**	**	*	9
Samancioglu et al. (2017)	**		*	*	**	**	*	9
Samara et al. (2017)	**		*	*	**	**	*	9
Arda Sürücü et al. (2018)	**		*	*	*	**	*	8
Tol et al. (2012)	**			*	*	**	*	7
Yacoub et al. (2011)	**			*	*	**	*	7

The Newcastle‐Ottawa Scale (NOS) for observational studies (Case–control, Cohort, and Cross‐sectional studies) was used (Wells et al., 2014) to critically appraise all included studies (see Supplementary File 1).

### 2.7 Synthesis

Data were analysed in three forms based on our objectives:

1. We performed a generic inverse variance analysis using the logarithmic odds ratio (LogOR) and SE to identify the association between glycaemic control and characteristics reported as associated with glycaemic control in bivariate analyses, such as age, gender, marital status, education, mental status, physical activity, smoking, obesity and self‐monitoring.
2. We used the Inverse Variance (I‐V) model to calculate the mean difference (MD) between adequate (HbA1c <7) and inadequate (HbA1c equal to or more than 7) levels of glycaemic control according to participants' characteristics such as age, Body mass index (BMI) and duration of diagnosis (ADA, 2021).
3. Moreover, we used the I‐V model to calculate the MD of HBA1c according to the studied groups [male vs. female, current smokers vs. non‐smokers, older (≥50 years) vs. younger aged (<50 years), obese (BMI ≥30 kg/m^2^) vs. non‐obese (BMI <30 kg/m^2^)] (World Health Organization, 2020).

Data analysis was performed using Review Manager Version 5.3 for windows.

### 2.8 Sensitivity analysis and publication bias

According to Egger and Smith (1998), publication bias assessment is relicable where ≥10 studies are pooled. Therefore, we assessed the publication bias using funnel plots and the Egger test. We performed a sensitivity analysis to ensure that none of the included studies affected the results and whether the overall effect size was statistically robust. We excluded one study in each scenario to determine the effect of each study on the overall effect size (Agency for Healthcare Research and Quality, 2014).

## 3 RESULTS

### 3.1 Study selection

**FIGURE 1:**
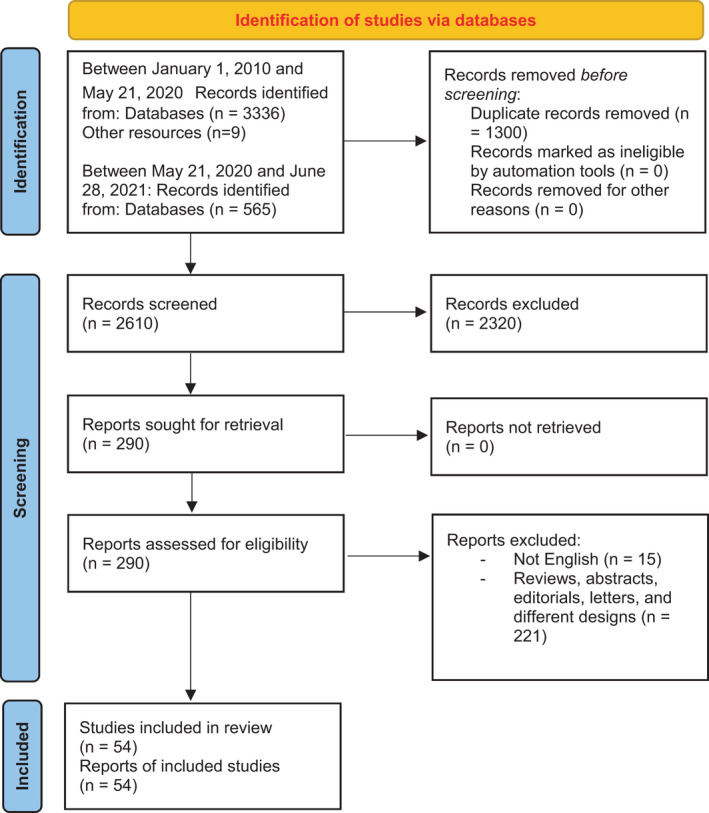
PRISMA flow diagram of included studies

The literature search of databases yielded 3345 records. Following the title/abstract and full‐text screening, 54 articles with a total of 41,079 participants were retained for inclusion in the systematic review. Of these, 32 articles reported adequate and relevant data for inclusion in the meta‐analysis. The flow of the study selection process is shown in Figure 1.

### 3.2 Study characteristics

Of the included studies, 17 articles were conducted in Saudi Arabia, seven each in Turkey and Iran, six in Jordan, three each in Iraq and Palestine, two each in Egypt, Bahrain, Kuwait and Oman, and one each in Lebanon, United Arab Emirates and Yemen. Forty‐nine studies were cross‐sectional, and five were cohort studies. Both genders were represented equally in the majority of the included studies. A total of 34 studies reported participants' level of education, with an average of 79.6% being university‐level educated. Insulin therapy was reported in 33 studies, on average used by 38.8% of participants. Obesity was reported in 47.6% of the participant population, and 25.5% were smokers. Uncontrolled diabetes (HbA1c >7%) was reported in 63% of the participants. Table 1 shows the baseline characteristics of the included studies and participants.

### 3.3 Quality of included studies

The quality of included studies, assessed using the NOS tool, ranged from good to very good for cross‐sectional studies, and was deemed very good for all included cohort studies participants (Table 2).

**TABLE 2:** The Newcastle‐Ottawa Scale (NOS) of Cross‐sectional studies and Cohort studies

Cohort studies									
Study ID	Selection				Comparability	Outcome			Quality score
	Representativeness of the exposed cohort	Selection of the non‐exposed cohort	Ascertainment of exposure	Demonstration that outcome of interest was not present at start of study	Comparability of cohorts on the basis of the design or analysis controlled for confounders	Assessment of the outcome	Was follow‐up long enough for outcomes to occur	Adequacy of follow‐up of cohorts	
Akin et al. (2019)	*			*	**	*	*	*	7
Almutairi & Alkharfy (2013)	*		*	*	**	*	*	*	8
Alzaheb & Altemani (2018)	*	*	*	*	**	*	*	*	9
Alzahrani et al. (2018)	*	*	*	*	**	*	*	*	9
Mansour et al. (2020)	*		*	*	**	*	*	*	8

Each (*) mean 1 on the score of NOS, and the final column count the total of these stars to give a final decision.

### 3.4 Characteristics of the participants linked to glycaemic control

#### 3.4.1 Older age (>50 years)

**FIGURE 2:**
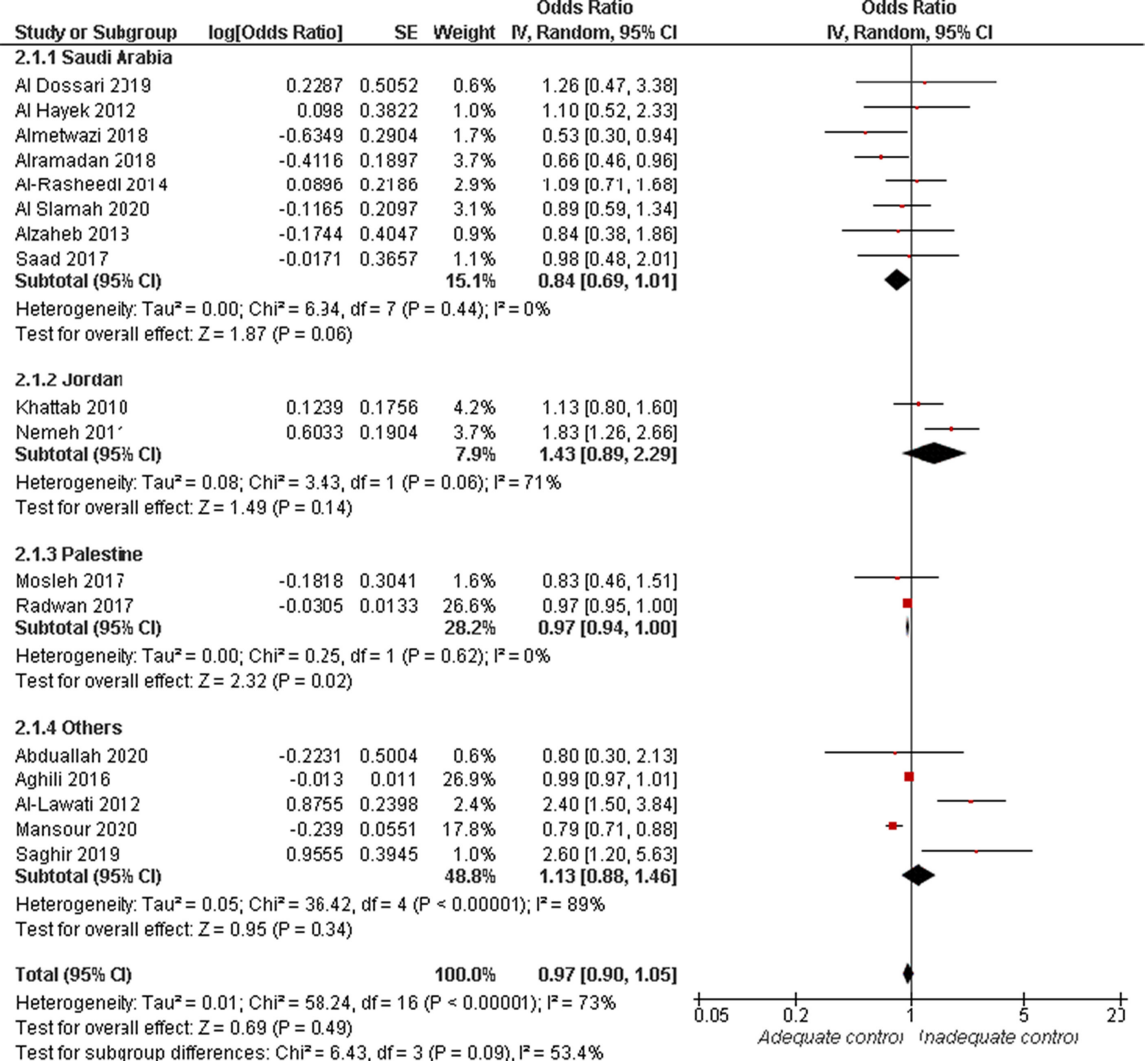
Forest plot of the odds ratio of age (>50 years) in relation to adequate glycaemic control

Overall effect estimates of 17 studies showed no significant association between risk of inadequate glycaemic control and older age (>50 years) (OR = 0.97, 95% CI: 0.90, 1.05; *p* = .49). Overall pooled data were heterogenous (*I* ^2^ = 73%, *p* < .0001). A subgroup analysis showed no significant association between risk of inadequate glycaemic control and older age in Saudi Arabia (OR = 0.84, 95% CI: 0.69, 1.01; *p* = .06) and Jordan (OR = 1.43, 95% CI: 0.89, 2.29; *p* = .14). In studies conducted in Palestine, older participants were associated with a lower risk of inadequate glycaemic control (OR = 0.97, 95% CI: 0.94, 1.00; *p* = .02). Pooled data in the subgroup analysis were homogenous (*I* ^2^ = 0%, *p* = .62), Figure 2. Moreover, participants with adequate glycaemic control were older than those with inadequate glycaemic control (MD = 0.70 years, 95% CI: 0.11, 1.29; *p* = .02), and the pooled data were homogenous (*I* ^2^ = 28%, *p* = .18), see Supplementary File 1, Figure A.

#### 3.4.2 Female gender

**FIGURE 3:**
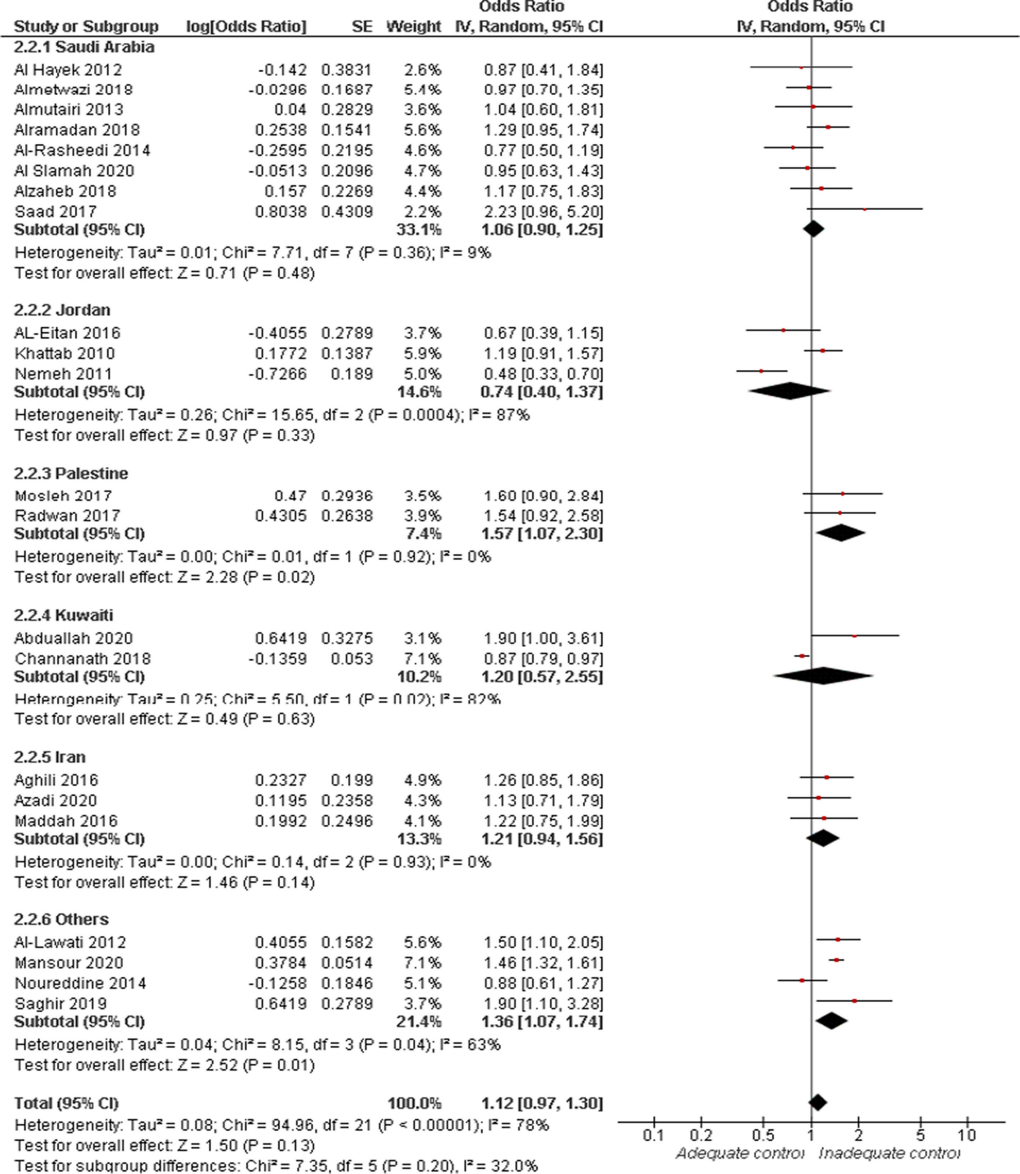
Forest plot of the odds ratio of inadequate glycaemic control by gender

Overall random‐effect estimates of 22 studies showed no significant association between risk of inadequate glycaemic control for female compared with male participants (OR = 1.12, 95% CI: 0.97, 1.30; *p* = .13). Overall pooled data were heterogenous (*I* ^2^ = 78%, *p* < .0001). Subgroup analysis showed no significant association between risk of inadequate glycaemic control in female compared with male participants in Saudi Arabia (OR = 1.06, 95% CI: 0.90, 1.25; *p* = .48), Jordan (OR = 0.74, 95% CI: 0.40, 1.37; *p* = .33), Kuwait (OR = 1.20, 95% CI: 0.57, 2.55; *p* = .63), and Iran (OR = 1.21, 95% CI: 0.94, 1.56; *p* = .14). For Palestine and other countries, female gender was associated with higher risk of inadequate glycaemic control (OR = 1.57, 95% CI: 1.07, 2.30; *p* = .02 and OR = 1.36, 95% CI: 1.07, 1.74; *p* = .01), respectively. Pooled data from Palestine were homogenous (*I* ^2^ = 0%, *p* = .92). The heterogeneity in the other subgroup was resolved by excluding the data from Noureddine et al. (2014) (*I* ^2^ = 0%, *p* = .65), with an effect estimate of (OR = 1.48, 95% CI: 1.34, 1.62; *p* < .0001), see Figure 3.

#### 3.4.3 Elevated WHR

**FIGURE 4:**
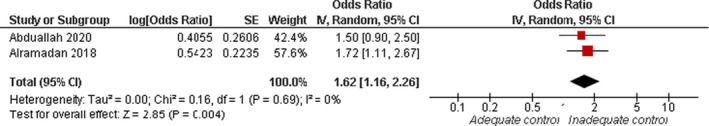
Forest plot of the odds ratio for inadequate glycaemic control in relation to elevated WHR

Pooled data of two studies demonstrated a significant elevation of the risk of inadequate glycaemic control in patients with elevated waist to hip ratio (WHR) (OR = 1.62, 95% CI: 1.16, 2.26; *p* = .004). Pooled data were homogenous (*I* ^2^ = 0%, *p* = .69), see Figure 4.

#### 3.4.4 Obesity

**FIGURE 5:**
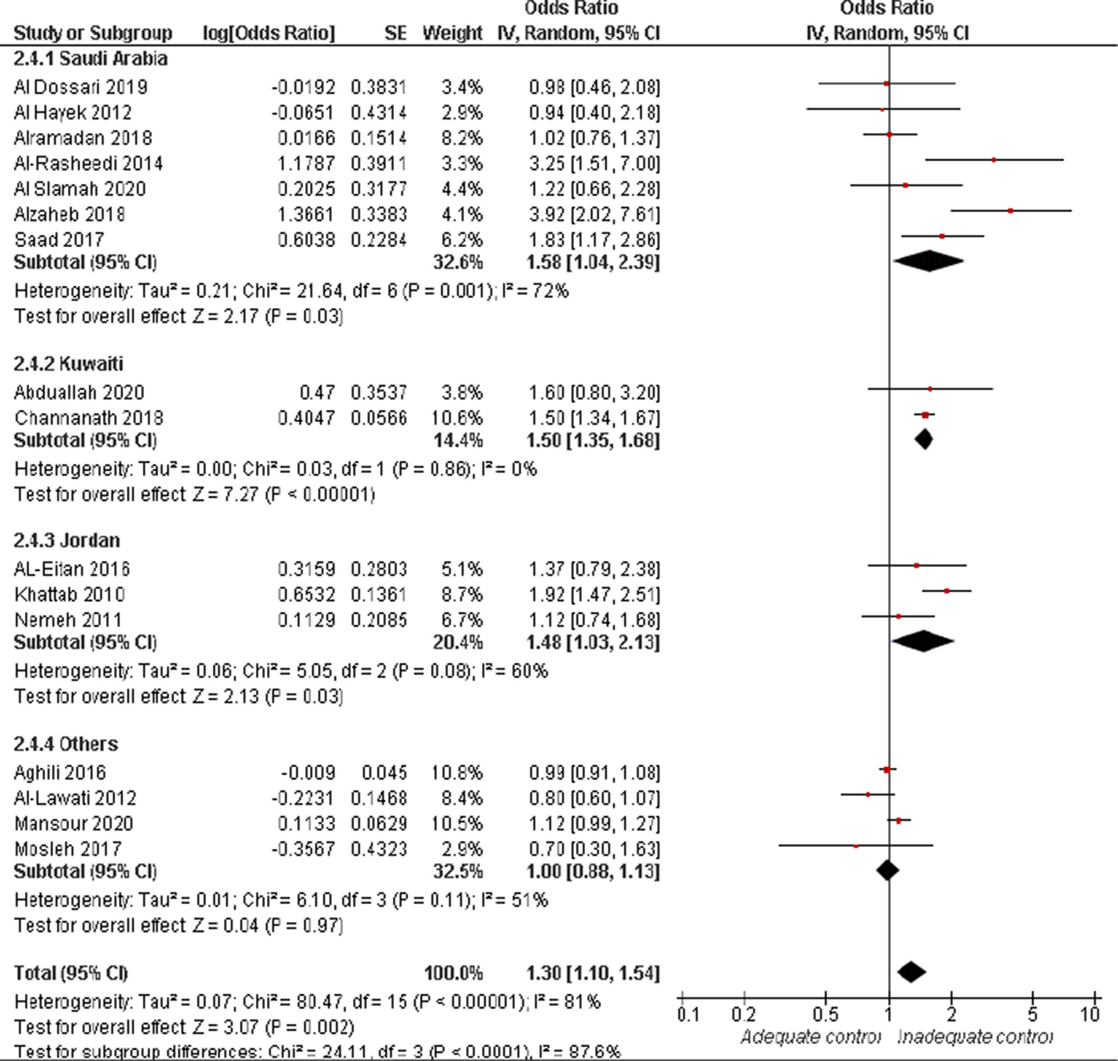
Forest plot of the odds ratio of inadequate glycaemic control in relation to obesity (BMI >30 kg/m^2^)

Overall random effect estimates of 16 studies showed a significantly increased risk of inadequate glycaemic control in obese patients (BMI >30 kg/m^2^) (OR = 1.30, 95% CI: 1.10, 1.54; *p* = .002). Overall pooled data were heterogenous (*I* ^2^ = 81%, *p* < .0001). Subgroup analyses showed a significant elevation in the risk of inadequate glycaemic control in obese participants in Saudi Arabia (OR = 1.58, 95% CI: 1.04, 2.39; *p* = .03), Jordan (OR = 1.48, 95% CI: 1.03, 2.13; *p* = .03) and Kuwait (OR = 1.50, 95% CI: 1.34, 1.67; *p* < .00001), see Figure 5. Moreover, participants with adequate glycaemic control had lower BMI compared with those with inadequate glycaemic control (MD = −1.16 kg/m^2^, 95% CI: −1.85, −0.47; *p* = .001), and the pooled data were heterogenous (*I* ^2^ = 73%, *p* < .0001). The heterogeneity was managed by excluding the data from Channanath et al. (2018) (*I* ^2^ = 47%, *p* = .06), see Supplementary File 1, Figure B. Furthermore, there was an association between glycaemic control and BMI, with lower HbA1c levels more likely in participants with lower BMI (<30 kg/m^2^) than participants with BMI >30 (MD = −0.32, 95% CI: −0.52, −0.12; *p* = .002). Pooled data were homogenous (*I* ^2^ = 5%, *p* = .35), see Supplementary File 1, Figure C.

#### 3.4.5 Smoking

**FIGURE 6:**
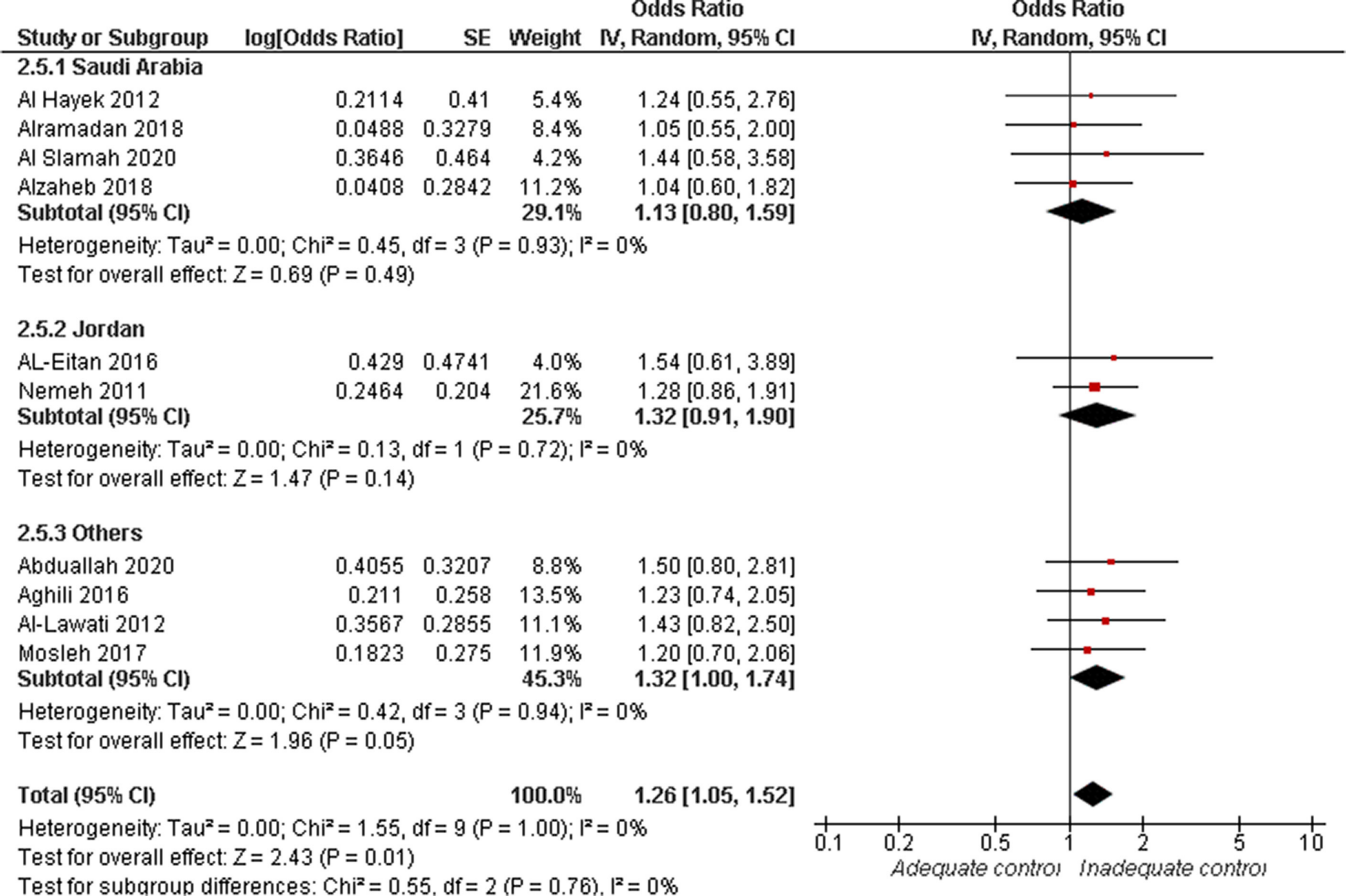
Forest plot of the odds ratio of inadequate glycaemic control by smoking status

Overall random‐effect estimates of 10 studies showed that the risk of inadequate glycaemic control was significantly elevated in smokers (OR = 1.26, 95% CI: 1.05, 1.52; *p* = .01). Overall pooled data were homogenous (*I* ^2^ = 0%, *p* = 1.00), see Figure 6.

#### 3.4.6 Duration of diabetes (>10 years)

**FIGURE 7:**
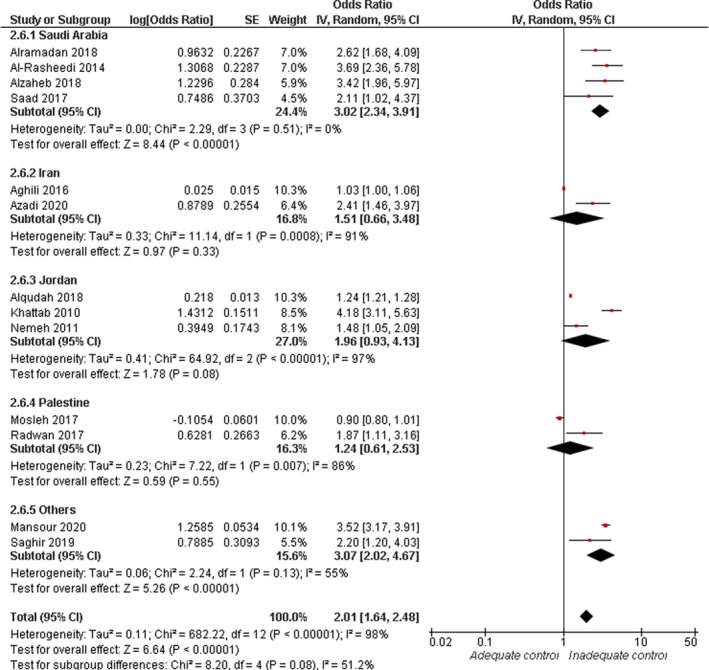
Forest plot of the odds ratio of inadequate glycaemic control in relation to extended duration of diabetes (>10 years)

Overall, random‐effect estimates of 13 studies showed an increased risk of inadequate glycaemic control in patients with longer duration of diabetes (OR = 2.01, 95% CI: 1.64, 2.48; *p* < .001). Overall pooled data were heterogeneous (*I* ^2^ = 98%, *p* < .001), see Figure 7. The heterogeneity could not be resolved by sensitivity analysis. Subgroup analysis demonstrated that in the Saudi population, the risk of inadequate glycaemic control was tripled in participants who had diabetes for more than 10 years compared with those who experienced the disease for a shorter duration (<10 years; OR = 3.02, 95% CI: 2.34, 3.91; *p* < .0001). Subgroup pooled data were homogenous (*I* ^2^ = 0%, *p* = .51). Moreover, participants with adequate glycaemic control tended to report shorter duration of diabetes (MD = −1.86, 95% CI: −3.55, −0.16; *p* = .03), see Supplementary File 1, Figure D.

#### 3.4.7 Self‐management

**FIGURE 8:**
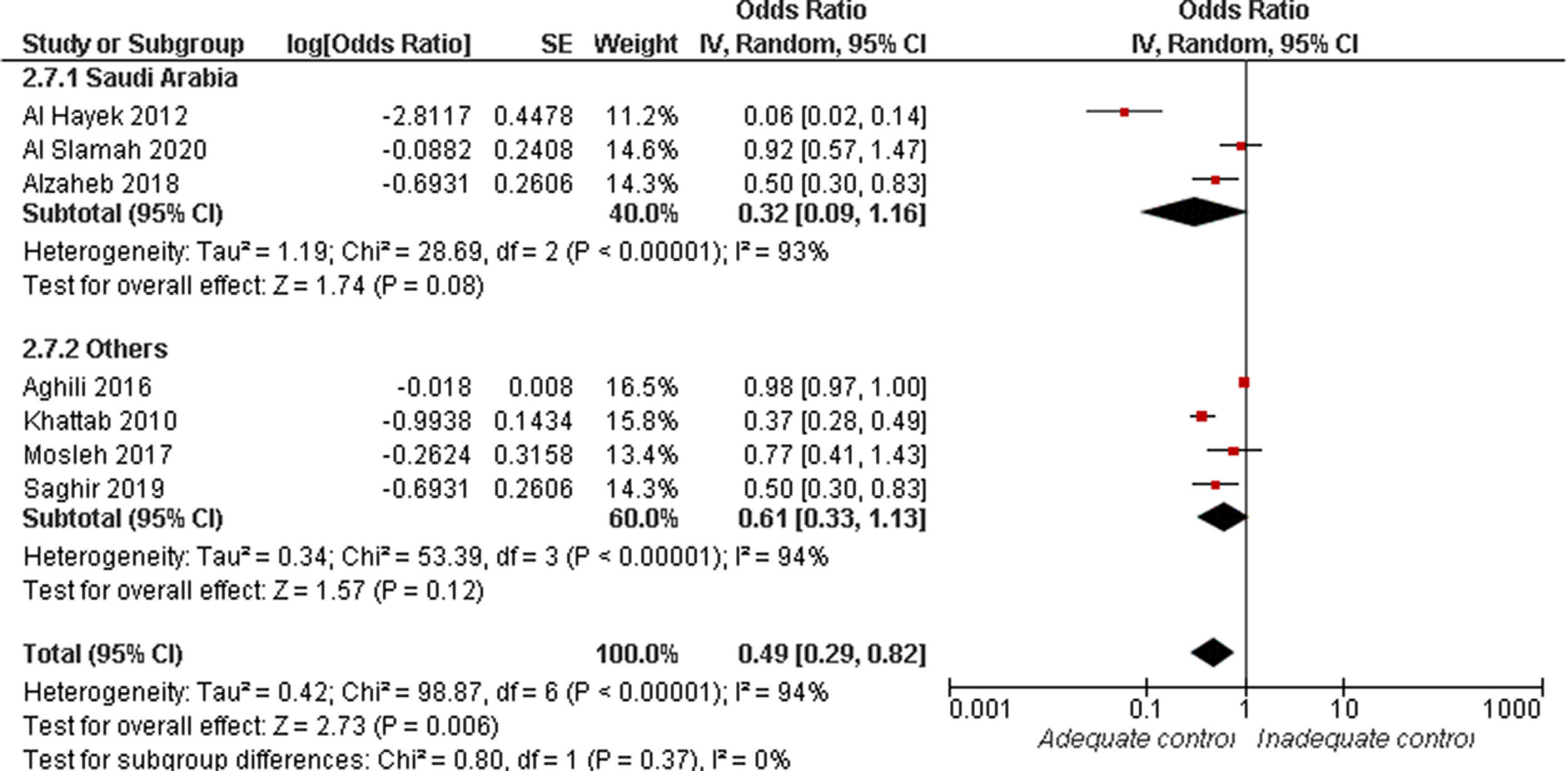
Forest plot of the odds ratio of inadequate glycaemic management in relation to self‐management

Pooled analysis of seven studies showed a significant association between lower risk of inadequate glycaemic control and self‐management (OR = 0.49, 95% CI: 0.29, 0.82; *p* = .006). Overall pooled data were heterogenous (*I* ^2^ = 94%, *p* < .001), see Figure 8. Heterogeneity was best resolved by excluding the data from (Aghili et al., 2016; Al‐Hayek et al., 2012; Khattab et al., 2010) (*I* ^2^ = 31%, *p* = .23), with the overall effect estimate still significant (OR = 0.65, 95% CI: 0.47, 0.89; *p* = .007).

#### 3.4.8 Physical activity

**FIGURE 9:**
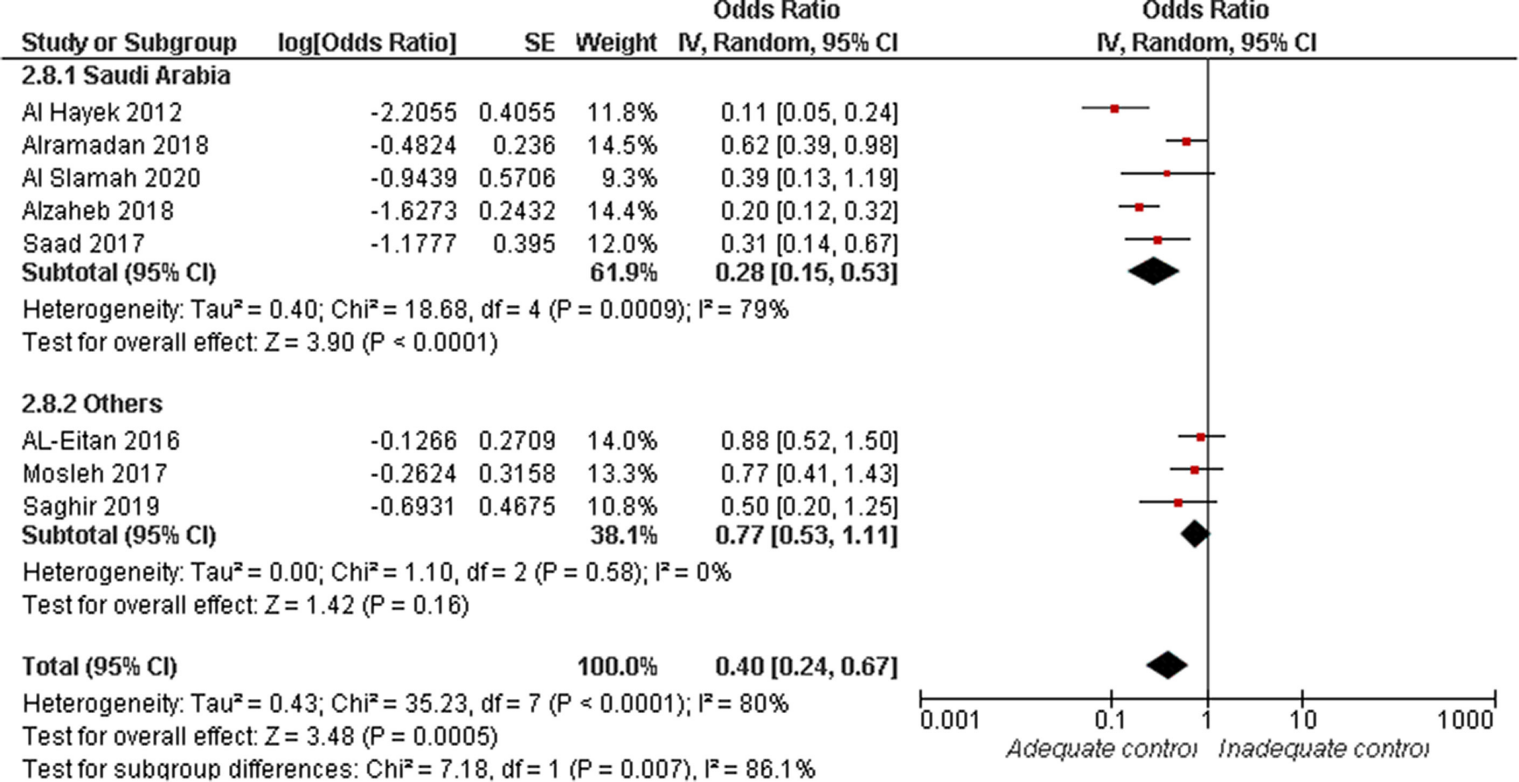
Forest plot of the odds ratio of inadequate glycaemic control in relation to physical activity

Overall random‐effect estimates of eight studies showed a significant association between the lower risk of inadequate glycaemic control and physical activity (OR = 0.40, 95% CI: 0.24, 0.67; *p* = .0005). Overall pooled data were heterogenous (*I* ^2^ = 80%, *p* < .0001), see Figure 9. Heterogeneity was best resolved by excluding the data from (AL‐Eitan et al., 2016; Alramadan et al., 2018; Mosleh et al., 2017) (*I* ^2^ = 49%, *p* = .10), with the overall effect estimate still significant (OR = 0.25, 95% CI: 0.15, 0.40; *p* < .0001).

#### 3.4.9 Diabetes knowledge

The pooled analysis of four studies showed no significant association between diabetes knowledge and glycaemic control (OR = 0.67, 95% CI: 0.39, 1.16; *p* = .15). Overall pooled analysis was heterogenous (*I* ^2^ = 81%, *p* = .001). Subgroup analysis showed no significant association in Saudi Arabia (OR = 0.55, 95% CI: 0.28, 1.07; *p* = .08) and other countries (OR = 0.89, 95% CI: 0.55, 1.42; *p* = .62), see Supplementary File 1, Figure E.

#### 3.4.10 Depression

Pooled analysis of three studies demonstrated no significant difference between depressed and non‐depressed participants in terms of glycaemic control (OR = 0.98, 95% CI: 0.93, 1.03; *p* = .42). Overall pooled data were homogenous (*I* ^2^ = 0%, *p* = .89). Subgroup analysis showed no significant association in Saudi Arabia (OR = 1.07, 95% CI: 0.74, 1.55; *p* = .73) and Iran (OR = 0.98, 95% CI: 0.93, 1.03; *p* = .39), see Supplementary File 1, Figure F.

#### 3.4.11 Anxiety

Data from three studies indicated no significant association between anxiety and glycaemic control (OR = 1.01, 95% CI: 0.99, 1.03; *p* = .53). Overall pooled data were homogenous (*I* ^2^ = 5%, *p* = .35). Subgroup analysis showed no significant association in Saudi Arabia (OR = 1.25, 95% CI: 0.89, 1.76; *p* = .20) and Iran (OR = 1.01, 95% CI: 0.98, 1.03; *p* = .59), see Supplementary File 1, Figure G.

#### 3.4.12 Employment

The overall fixed‐effect showed no significant association between employment and glycaemic status (OR = 1.03, 95% CI: 0.85, 1.24; *p* = .75). Overall pooled data were homogenous (*I* ^2^ = 19%, *p* = .28). Subgroup analysis showed no significant association in Saudi Arabia (OR = 0.95, 95% CI: 0.75, 1.19; *p* = .63) and other countries (OR = 1.23, 95% CI: 0.89, 1.72; *p* = .21), see Supplementary File 1, Figure H.

#### 3.4.13 Marital status

The overall random‐effect estimate demonstrated no significant association between marital status and glycaemic control (OR = 1.07, 95% CI: 0.85, 1.35; *p* = .55). Overall pooled data showed minor heterogeneity (*I* ^2^ = 38%, *p* = .09). Subgroup analysis showed no significant association in Saudi Arabia (OR = 1.06, 95% CI: 0.68, 1.67; *p* = .79), Iran (OR = 0.96, 95% CI: 0.72, 1.27; *p* = .76), Jordan (OR = 1.30, 95% CI: 0.92, 1.84; *p* = .14), and Palestine (OR = 0.84, 95% CI: 0.24, 2.96; *p* = .79), see Supplementary File 1, Figure I.

## 4 DISCUSSION

Previous meta‐analyses focused on the relationship between individual factors and the development of diabetes (Lustman et al., 2000; Rodríguez‐Gutiérrez & Montori, 2016); however, this review is the first to examine a broad spectrum of elements regarding their possible influence on glycaemic control in participants with established T2DM in the MENA region. This systematic review and meta‐analysis found no significant association between glycaemic control and age, gender, diabetes knowledge, depression, anxiety, employment or marital status. However, in Palestine, older participants and females had a lower risk of inadequate glycaemic control. Overall, a greater risk of inadequate glycaemic control was seen in participants with obesity, higher WHR, smokers and those who had lived with diabetes for a longer duration (>10 years). Studies from Saudi Arabia demonstrated those with obesity and longer duration with diabetes at greater risk of inadequate control, as were participants from Jordan and Kuwait with obesity. Moreover, those with better self‐management and greater physical activity had a lower risk of inadequate glycaemic control.

Regarding the effect of demographic factors on the development of inadequate glycaemic control, our findings demonstrated a significant difference in age between participants with adequate and inadequate glycaemic control (MD = 0.70 years, *p* = .020). A cross‐sectional analysis of 1438 participants by Berkowitz et al. (2013), reported that young age patients were significantly associated with inadequate glycaemic control (*p* < .001). Similarly, a nationwide cross‐sectional study of 697 American patients with T2DM showed that adequate glycaemic control was prevalent among older patients. Moreover, these authors suggested that young patients undergo HbA1c assessments at least annually (Comellas et al., 2019). One suggested explanation for these observations is that older age is associated with a reduction in the lifespan of the red blood corpuscles, reducing their exposure to circulating glucose, thereby lowering blood levels of HbA1c (Dubowitz et al., 2014; Shperling & Danon, 1990). However, the overall odds ratio in our study showed no association between these variables. Similarly, Wiener & Roberts (1999) found no significant correlation in 399 participants between age and HbA1c levels. The lack of significance was ascribed to the exclusion of participants who had impaired glucose tolerance yet were eligible for inclusion in the normoglycaemic group based on a fasting plasma glucose value <6.4 mmol/L, a criterion that does not completely rule out diabetes in all participants, particularly non‐insulin‐independent diabetes.

Subgroup analysis surprisingly showed a significant inverse relationship between age and HbA1c among Palestinian participants. This relationship can perhaps be attributed to differences in attitudes among young participants compared with their elders. Younger individuals are often regarded or observed to be more negligent of the hazards of diabetes and the necessary self‐management activities to control their glucose levels; conversely, older participants tend to be more careful and adherent to their treatment regimen (Toh, 2011). Radwan et al. (2018) found a significant association between old age and adequate glycaemic control (OR = 0.97; 95% CI: 0.945, 0.995). Their results contradict previous findings because of differences in the demographics of the studied populations, especially in terms of the age distribution (Radwan et al., 2018). Higher percentages of participants were of older age in the Palestinian samples than in other study groups, with 34% of participants >61 years (Radwan et al., 2018) and more than 50% ≥58 years (Mosleh et al., 2017). However, studies from Aldossari et al. (2019), Nemeh et al. (2011), and Saghir et al. (2019) providing data from Saudi Arabia, Jordan, and other locations, respectively, did not show a significant inverse correlation between age and HbA1c. This could be attributed to the lower proportion of people >60 years in these studies [10.5% in Aldossari et al. (2019), 26% in Nemeh et al. (2011), and 25% in Saghir et al. (2019)].

The second demographic aspect examined was gender. No significant correlation was seen between gender and the risk of inadequate glycaemic control. This finding is inconsistent with a previous patient‐level pooled analysis of six RCTs, totalling 2600 participants with diabetes, which found women more prone to hypoglycaemia and high HbA1c levels (Kautzky‐Willer et al., 2015). Another systematic review and meta‐analysis from 2015 found that the risk of inadequate glycaemic control was higher in women; however, three of their included studies were deemed unreliable due to the high risk of bias (Sobers‐Grannum et al., 2015). Various explanations have been put forward for these observations. It has been proposed that women show lower levels of adherence to medications, to which they more frequently report side effects (Thunander Sundbom & Bingefors, 2012). Differences in lifestyles (e.g. exercise) as well as in psychosocial perceptions might also play a role in gender‐based differences in glycaemic control (Walker et al., 2006). Socio‐economic differences might also play a part; however, these were not considered in any study. Differences in physiology have also been suggested to underlie any sex‐based differences in HbA1c levels; however, further investigation is required of any sex‐based differences in efficacy/treatment response, as current evidence for factors such as dissimilarities in body composition is unlikely to provide sufficient explanation for such observations (Radwan et al., 2018). In Lebanon, Noureddine et al. (2014) found no association between gender and the risk of T2DM within the Lebanese population (Noureddine et al., 2014). Similarly, in Saudi Arabia, Al‐Rasheedi (2014) failed to demonstrate a significant connection between the two factors (Al‐Rasheedi, 2014). However, though insignificant, both studies showed that the percentage of males within the inadequate glycaemic control group was higher than in the adequate glycaemic control group.

With regard to the anthropometric measures, this review found a significant association between increased WHR and high HbA1c levels (*p* < .05). Such results are congruent with the findings from (Vazquez et al., 2007), where 32 different populations from 29 studies were analysed. In that study, WHR was found to correlate well with the incidence of diabetes. WHR is considered by some a reliable measure of visceral fat (Gadekar et al., 2020). An elevated WHR indicates greater accumulation of visceral fat stores, a feature that negatively influences the metabolism of hormones such as insulin through free fatty acid secretion, which hinders insulin uptake by the liver leading to hyperinsulinemia and subsequent insulin resistance (Despres et al., 1995; Kahn & Flier, 2000). Nevertheless, reports have questioned the reliability of WHR for accurately reflecting changes in visceral fat (Van Der Kooy et al., 1993). This suggests that more sensitive measures should be used to properly explore the relationship between fat mass and glycaemic control.

Obesity, signified by BMI >30 kg/m^2^, was significantly associated with an increased risk of inadequate glycaemic control. This observation supports the findings of Abdullah et al. (2020). Moreover, a significant association was detected between BMI and HbA1c levels. As mentioned above, obesity (represented by both high WHR and BMI) is usually accompanied by insulin resistance and insensitivity due to the effect of visceral fat on insulin secretion and clearance. Accordingly, this sheds some light on a possible association between insulin resistance itself and HbA1c. Saha and Schwarz (2017) found HbA1c a reliable tool for predicting insulin resistance and inadequate glycaemic control among 3578 normoglycaemic and hyperglycaemic individuals (Saha & Schwarz, 2017).

The third category of factors related to glycaemic control is that of lifestyle. This includes physical activity, smoking, and psychological and social patterns. This review indicated a significant association between smoking and inadequate glycaemic control. Other meta‐analyses have found significant associations between smoking (whether active or passive) and the risk of developing T2DM (Pan et al., 2015; Soulimane et al., 2014; Wang et al., 2013). With the exception of Alzaheb and Altemani (2018), all the included studies that analysed smoking used HbA1c as an index for glycaemic control, and reported significant positive correlations between smoking and HbA1c. Numerous explanations have been put forward to explain the relationship between smoking and glycaemic control. One theory proposed that smoking raises HbA1c levels by altering the erythrocytic membrane permeability to glucose, consequently increasing haemoglobin glycation (Higgins et al., 2009). Moreover, smoking lowers oxygen saturation in the blood, which leads to higher levels of deoxyhemoglobin, which is more readily glycated than oxyhemoglobin, accelerating HbA1c formation. Other possible explanations pertain to the amplifying effect exerted by smoking on the levels of 2,3‐diphosphoglycerate, an intermediate of glycolysis that correlates with high levels of HbA1c. Furthermore, one of the components of tobacco smoke, carbon monoxide, increases the lifespan of circulating erythrocytes, increasing their blood glucose exposure (Beutler, 1975; Smith et al., 1982). The strong association between smoking and T2DM may offer a new approach for avoiding a rise in HbA1c levels, and therefore reducing the incidence of the disease. For example, awareness campaigns could educate people living with diabetes regarding the particular seriousness of smoking and its harmful influence on their glycaemic status.

Regarding physical activity, this review found that increased physical effort led to a significant reduction in the risk of inadequate glycaemic control (*p* < .001). Similarly, Aune et al. (2015) presented a meta‐analysis of 87 studies (including three RCTs and 84 cohorts) demonstrating an inverse relationship between total physical activity (i.e. leisure‐time, occupational and transport) and the risk of inadequate glycaemic control (Aune et al., 2015). The consequential reduction in body adiposity, a major contributing factor to the development of diabetes, can explain this response to sustained, regular physical activity (Mozaffarian et al., 2011; Rana et al., 2007). Alternatively, Aune et al. (2015) pointed out that physical exercise involves concentric contractions of the body's skeletal muscles, which take up glucose from the circulation (Aune et al., 2015). This process is mediated through the GLUT4 glucose transporter located on the membranes of striated muscle cells. Studies have reported that physical activity enhances GLUT4 translocation on the myocytic membrane, thereby increasing the rate of glucose uptake by muscles and improving glucose homeostasis (Colberg et al., 2010; Perseghin et al., 1996; Röckl et al., 2008). On sensitivity analysis, three records were excluded to resolve the observed heterogeneity (AL‐Eitan et al., 2016; Alramadan et al., 2018; Mosleh et al., 2017). The first two of these observed no significant association between physical exercise and glycaemic control, which AL‐Eitan et al. (2016) ascribed to possible selective information sharing by the participants during their interviews, commonly known as reporting bias, resulting in the inconsistent findings of studies in this review (AL‐Eitan et al., 2016).

Another factor frequently included in lifestyle‐modification interventions is self‐management (Ali et al., 2012; Muchiri et al., 2016; Zheng et al., 2019). In the analysis by Garcia‐Molina et al., 16 studies introduced self‐management as part of their intervention. These observations emphasise the importance of implementing lifestyle changes to achieve better glycaemic control. García‐Molina et al. (2020) found, analysing the findings of 28 studies, that lifestyle interventions were more efficient than standard care in reducing the risk of inadequate glycaemic control (García‐Molina et al., 2020). Heterogeneity in this analysis was best resolved by excluding (Aghili et al., 2016; Al‐Hayek et al., 2012; Khattab et al., 2010). Aghili et al. (2016) noted a negative relation between self‐care practices and HbA1c levels, ascribed to the strong association between self‐management and diabetes‐related distress. The latter has been associated with negative responses (e.g. increasing depressive symptoms linked to reducing adherence to medication), directly and indirectly leading to uncontrolled glucose levels (Aikens, 2012; Gonzalez et al., 2008). Khattab et al. (2010) and Al‐Hayek et al. (2012) used self‐report to collect data concerning participants' medication adherence, glucose self‐monitoring and dietary‐intake (i.e. self‐management practices), which risked recall bias, a type of error that might have led to exaggerated findings (Al‐Hayek et al., 2012; Khattab et al., 2010).

Lastly, a highly significant link was demonstrated between longer disease duration and inadequate glycaemic control. By contrast, a UK‐based cross‐sectional study found an inverse relation between the two variables. This finding may be attributed to the different healthcare systems of developing and developed countries. Abubakari et al. (2016) hypothesized that longer disease duration allows participants to better understand their condition's nature, consequences and responsibilities, which assists in the management of negative emotions that might affect their response, allowing them to better control their disease (Abubakari et al., 2016).

### 4.1 Strengths and limitations

There are several limitations to this meta‐analysis. First, most included studies used self‐report to collect outcome data, potentially leading to recall bias, which might either exaggerate or underestimate the true effect size. Second, in terms of the anthropometric markers, only two measures were observed, WHR and BMI. These measurements have been reported as less informative measures of obesity than other measurements such as waist circumference and the waist‐to‐height ratio (Rajput et al., 2014; Vazquez et al., 2007). Third, the high heterogeneity observed between studies was not always resolvable, potentially indicating diversity in the methodology or baseline criteria of the included studies. Greater consistency in design among studies is recommended for the future. Fourth, we only included English language studies, and peer‐reviewed published articles to ensure the quality of the included studies' methodologies.

The strengths of this review include the relatively large number of included studies, adding to the strength of association between the predictors of glycaemic control.

In conclusion, the current evidence suggests an increased risk of inadequate glycaemic control in patients with elevated WHR, longer disease duration, obesity and smokers, with lower risk of inadequate control associated with physical activity and self‐management. Further longitudinal studies are required to better understand these variations and to assess all predictors of glycaemic control in participants with T2DM, and to further provide a strong basis for future recommendations to optimize glycaemic control.

## 5 RECOMMENDATIONS

Review findings illustrated some variability in variables which exert significant impact across the countries of the Middle East and North Africa. Irrespective of whether they are middle‐income or high‐income, countries should use national and regional data to inform the development of strategies sensitive to local and global influences. Broadly, however, policymakers and clinicians should pay attention to spreading awareness campaigns to educate people living with diabetes regarding the seriousness and implications of obesity and smoking for glycaemic status. Regular exercise is essential, especially in people with living with diabetes for long periods, who should be encouraged to follow a balanced physical program to improve their glycaemic status. Diabetes education and self‐care management should be integral to the management of all people living with diabetes regardless of their medication and management pathways.

## CONFLICT OF INTEREST

None.

6

### PEER REVIEW

The peer review history for this article is available at https://publons.com/publon/10.1111/jan.15255.

## Supporting information


Appendix S1

Appendix S2
