# Supplementary material for: Glycaemic control and its associated factors in patients with type 2 diabetes in the Middle East and North Africa: An updated systematic review and meta‐analysis
Source: J Adv Nurs. 2022 May 27;78(8):2257–76. doi: 10.1111/jan.15255 (PMC9541219; doi:10.1111/jan.15255)
Supplement: Supplementary file 1 — Appendix S1 [file JAN-78-2257-s001.docx]

**Quality appraisal:** The Newcastle-Ottawa Scale (NOS) for observational studies (Case-control, Cohort, and Cross-sectional studies) was used [1]. The NOS uses a star system (with a maximum of 9 stars) to evaluate a study in 3 domains: selection of participants (4 items; Representativeness of the sample, Selection of the Non-Exposed, Ascertainment of Exposure, Non-respondents); comparability of study groups (2 items; Control for Confounders); and the ascertainment of outcomes of interest (3 items; Assessment of outcome, Was Follow-Up Long Enough for Outcomes to Occur, Adequacy of Follow-Up of Cohorts) (Table 2). We interpreted the score as follows: very good studies (9 points), good studies (7-8 points), satisfactory studies (5-6 points), unsatisfactory studies (0-4 points) [2].

**Assessment of Heterogeneity:** Forest plots were inspected visually for any heterogeneity. We assessed the heterogeneity using the I-square (I^2^) and Chi-square (Chi^2^) tests. For the Chi^2^ test of the Q-statistics, an alpha level below 0.1 was considered significant heterogeneity [3]. To quantify the magnitude of heterogeneity among effect estimates of the included studies, we interpreted the I^2^ test results as the follows: values from 0% to 30% indicated likely minimal heterogeneity; values from 30% to 60%, likely moderate, and values from 60% to 100%, likely substantial heterogeneity [3].

**Search term**

| **Concept** | **Term** |
| --- | --- |
| **#1** | ((((((diabetes mellitus, type 2[MeSH Terms]) OR (diabetes mellitus, type 2[Title/Abstract])) OR (Type 2 Diabetes[Title/Abstract])) OR (Non-insulin dependent diabetes[Title/Abstract])) OR (adult onset diabetes[Title/Abstract])) OR (Type ii diabetes[Title/Abstract])) OR (NIDDM[Title/Abstract]) |
| **#2** | ((((((((((Glycemic Control[Title/Abstract]) OR (Glycaemic control[Title/Abstract])) OR ("Glycemic Control"[Title/Abstract])) OR (Blood Glucose[Title/Abstract])) OR (Blood Glucose Self-Monitoring[Title/Abstract])) OR (Glycated Hemoglobin A[Title/Abstract])) OR (Hemoglobin A1c[Title/Abstract])) OR (HBA1c[Title/Abstract])) OR (Blood Glucose[MeSH Terms])) OR (Blood Glucose Self-Monitoring[MeSH Terms])) OR (Glycated Hemoglobin A[MeSH Terms]) |
| **#3** | ((((((((((((((((Middle East[Title/Abstract]) OR (Jordan[Title/Abstract])) OR (UAE[Title/Abstract])) OR (Bahrain[Title/Abstract])) OR (Iraq[Title/Abstract])) OR (Saudi Arabia[Title/Abstract])) OR (Kuwait[Title/Abstract])) OR (Yemen[Title/Abstract])) OR (Iran[Title/Abstract])) OR (Syria[Title/Abstract])) OR (Israel[Title/Abstract])) OR (Oman[Title/Abstract])) OR (Palestine[Title/Abstract])) OR (Qatar[Title/Abstract])) OR (Lebanon[Title/Abstract])) OR (Egypt[Title/Abstract])) OR (Turkey[Title/Abstract]) |
| **#4** | **#1 AND #2 AND #3** |
| **#5** | **#4 Limited to "Last 10 years" and "English"** |

**Figure A: Forest plot of the mean difference between adequate and inadequate glycemic control in terms of participants' age**

**
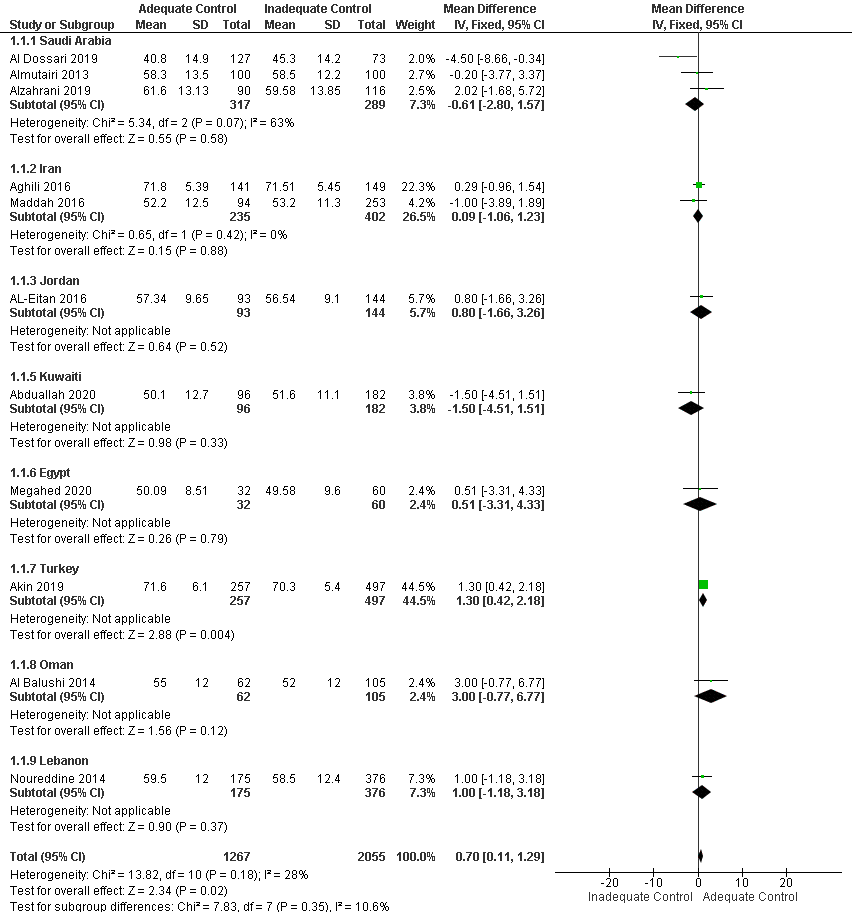
**

**Figure B: Forest plot of the mean difference between adequate and inadequate glycemic control in terms of BMI values**

**
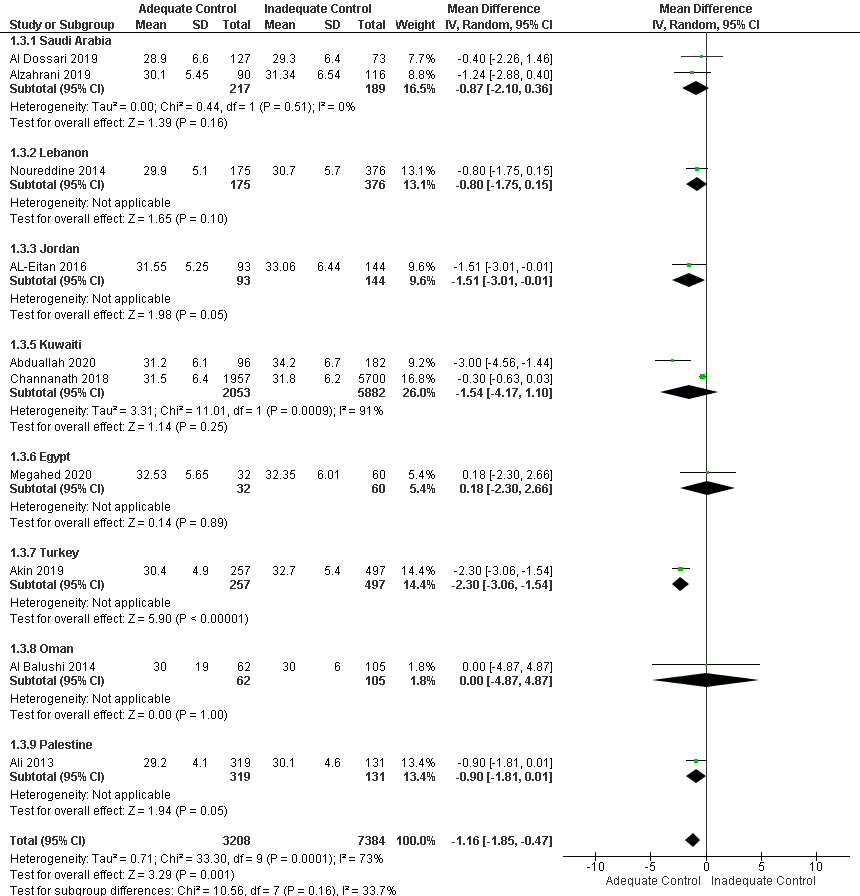
**

**Figure C: Forest plot of the mean difference in HbA1c values between obese and non-obese participants**

**
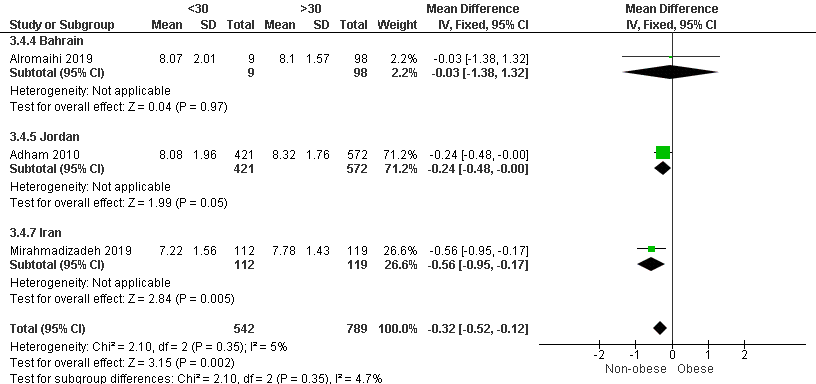
**

**Figure D: Forest plot of the mean difference between adequate and inadequate glycemic control in terms of diabetes duration in years**

**
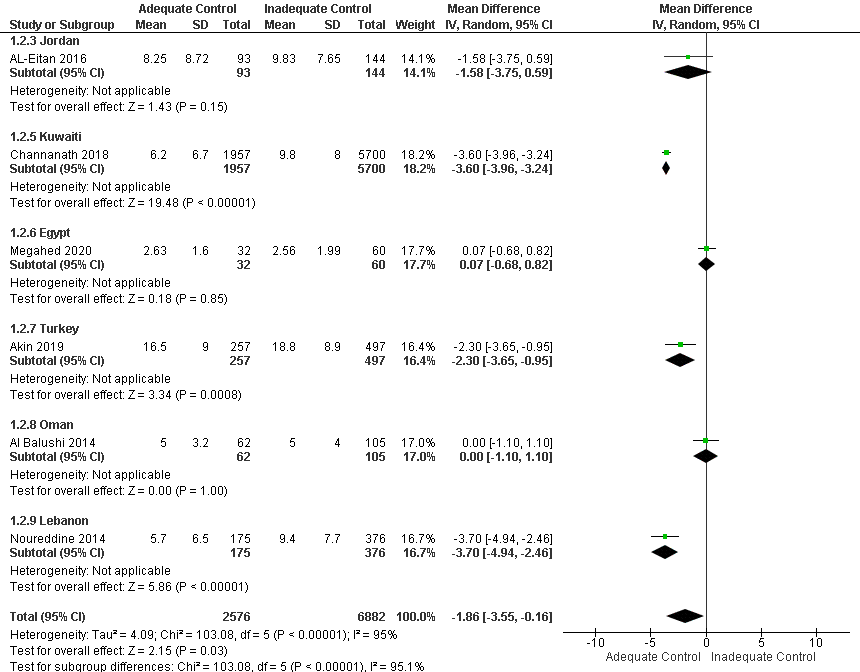
**

**Figure E: Forest plot of the Odds Ratio for inadequate glycemic control in relation to diabetes knowledge**

**
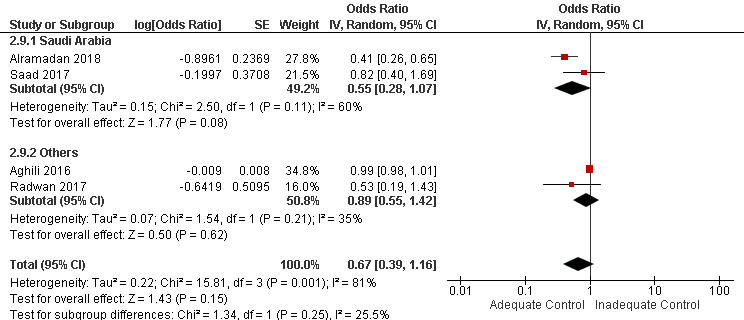
**

**Figure F: Forest plot of the Odds Ratio of inadequate glycemic control in relation to depression**

**
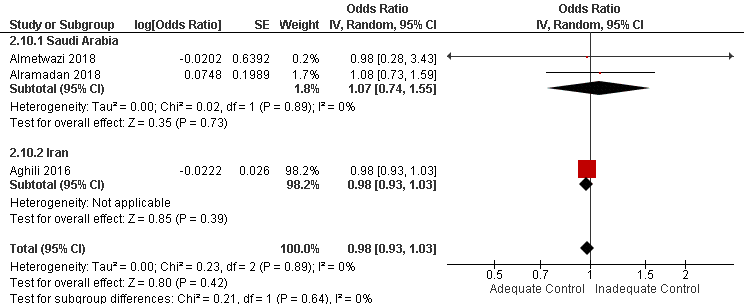
**

**Figure G: Forest plot of the Odds Ratio for inadequate glycemic control in relation to anxiety**

**
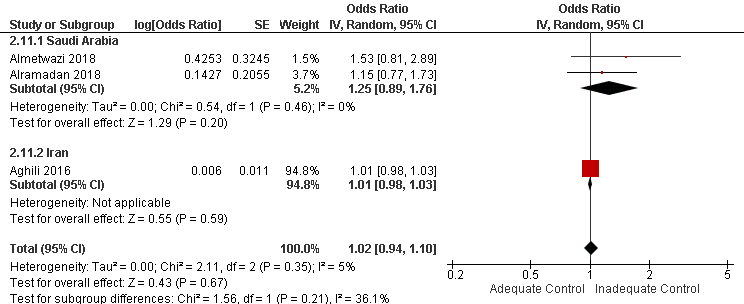
**

**Figure H: Forest plot of the Odds Ratio for inadequate glycemic control in relation to employment**

**
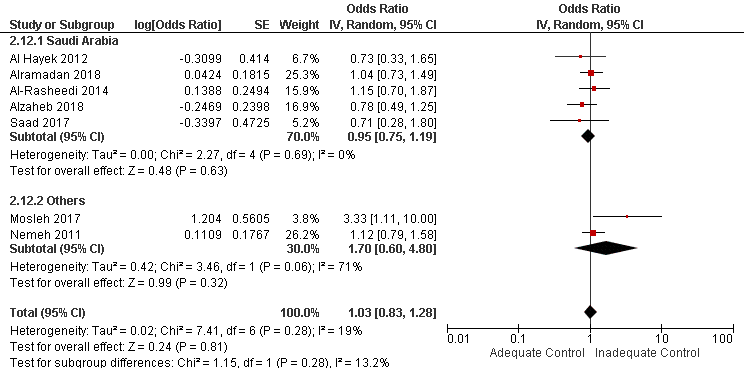
**

**Figure I: Forest plot of the Odds Ratio for inadequate glycemic control by marital status**

**
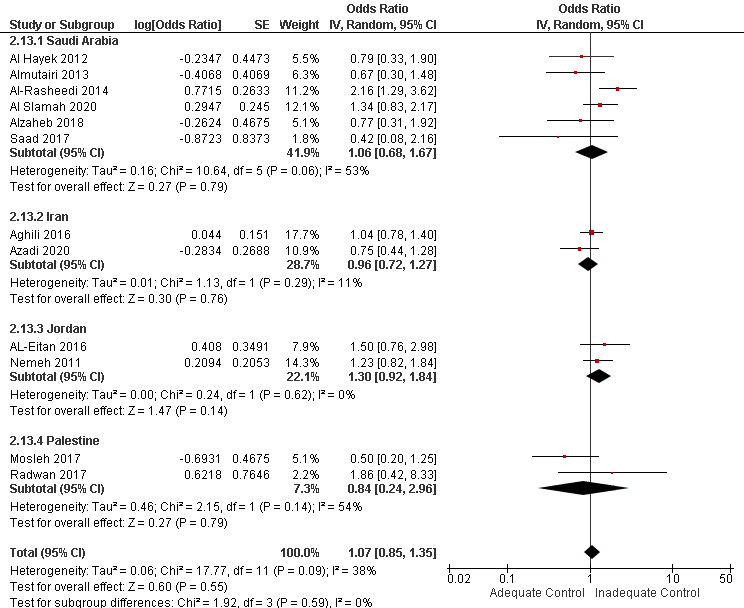
**

References

1. Wells G, Shea B, O’Connell D, Peterson J, Welch V, Losos M, Tugwell P. Newcastle-Ottawa quality assessment scale cohort studies. Visual Communication Quarterly. 2014.

2. Modesti P, Reboldi G, Cappuccio F. NEWCASTLE-OTTAWA QUALITY ASSESSMENT SCALE (adapted for cross sectional studies). PLoS One. 2016; : 1–2.

3. Higgins JPT, Thomas J, Chandler J, Cumpston M, Li T, Page MJ, Welch VA. Cochrane Handbook for Systematic Reviews of Interventions [Internet]. Cochrane Handbook for Systematic Reviews of Interventions. Wiley; 2019. Available from: https://onlinelibrary.wiley.com/doi/book/10.1002/9781119536604
