# Supplementary material for: Glycaemic control and its associated factors in patients with type 2 diabetes in the Middle East and North Africa: An updated systematic review and meta‐analysis
Source: J Adv Nurs. 2022 May 27;78(8):2257–76. doi: 10.1111/jan.15255 (PMC9541219; doi:10.1111/jan.15255)
Supplement: Supplementary file 2 — Appendix S2 [file JAN-78-2257-s002.docx]

| Study ID | Study title | Country | Sample size | Age | Gender | Educated | Use of insulin (%) | Disease duration | % of uncontrolled Glycemic | BMI | HbA1c (%) | Fasting plasma glucose (mmol/L) | Obesity | Smoking |
| --- | --- | --- | --- | --- | --- | --- | --- | --- | --- | --- | --- | --- | --- | --- |
| Abdullah 2019 | Glycemic control in Kuwaiti diabetes patients treated with glucose-lowering medication | Kuwait | 278 | 51.1 (11.7) | M: 40%  F: 60% | NA | 45.7% | NA | 65.5% | 33.1 (6.7) | 8.17 (2.24) | 9.42 (4.26) | 68.2% | 11.2% |
| Abuhegazy 2011 | Depression and glycemic control in a sample of patients with type 2 diabetes | Egypt | 180 | 51.2 (6.1) | M:180 | 64.4% | NA | 10.8 (3.6) | NA | 31.7 (5.9) | 8.7 (2.04) | 179.6 (7.6) | NA | 44.4% |
| Adham 2010 | Glycaemic control and its associated factors in type 2 diabetic patients in Amman, Jordan | Jordan | 1000 | 58.1 (9.3) | M:49.5%  F: 50.5% | 90% | 45.6% | 9.4 (7.2) | 75.9% | NA | 8.1 (1.8) | NA | 57.6% | 15% |
| Aghili 2016 | Type 2 Diabetes: Model of Factors Associated with Glycemic Control | Iran | 380 | 54.73 (8.00) | M:46.6%  F:53.4% | 100% | 43.4% | 8.94 (6.57) | NA | 28.3 (4.30) | 7.78 (1.7) | NA | NA | 13.7% |
| Ahmadian 2018 | The Relation between Anxiety, Depression and Sexual Dysfunction and the Level of Blood Glucose Control in Patients with Type 2 Diabetes Attending Endocrine Clinic of Taleghani Hospital | Iran | 141 | NA | NA | NA | NA | NA | 48.2% | NA | NA | NA | NA | NA |
| Akin 2019 | Overtreatment and Hypoglycemia Prevalence In Geriatric Patients With Type-2 Diabetes In The Turkish Population | Turkey | 755 | 70.9 (5.9) | M: 38.3%  F: 60.67 | NA | 65.8% | M: 18.3 (8.5) | 65.9% | 32.7 (5.4) | M: 7.8 (1.6)  F: 7.6 (1.4) | NA | NA | NA |
|  |  |  |  |  |  |  |  | F: 18.7 (9.1) |  |  |  |  |  |  |
| Al Hayek 2012 | Association between diabetes self-care, medication adherence, anxiety, depression, and glycemic control in type 2 diabetes | Saudi Arabia | 147 | 57.3 (14.4) | M: 67.3%  F: 32.7% | NA | NA | 12.7 (7.3) | 83.4% | NA | <7%: 29.3% | NA | 22.4% | 28.6% |
|  |  |  |  |  |  |  |  |  |  |  | >7%: 70.7 |  |  |  |
| Al Saweer 2015 | The Profile of Vitamin D among Type 2 Diabetes Mellitus Patients | Bahrain | 268 | 57.6 (10.6) | M: 43%  F: 57% | 94.55 | NA | 8.5 (12.08) | NA | NA | NA | NA | NA | NA |
| Al Slamah 2020 | Correlates of type 2 diabetes and glycaemic control in adults in Saudi Arabia a secondary data analysis of the Saudi health interview survey | Saudi Arabia | 808 | 38.38 (16.1) | M: 59%  F: 41% | 40.8% | NA | NA | 41% | NA | NA | NA | 86.2% | 14.1% |
| ALAboudi 2016 | Self-efficacy, self-care behaviours and glycaemic control in type 2 diabetic patients in Riyadh, Saudi Arabia | Saudi Arabia | 75 | 54 (9.2) | M:77.3%  F:22.7% | 100% | 37.4% | 12.6 (8.4) | 96% | NA | 9.26 (1.68) | NA | NA | NA |
| Al Balushi  2014 | Glycemic control among patients with type 2 diabetes at a primary health care center in Oman | Oman | 177 | 53 (12) | M: 40%  F: 60% | NA | 11% | 5.0 (2.3-7.0) | 65% | 30 (12) | NA | NA | NA | NA |
| Albasheer 2017 | Depression and related risk factors among patients with type 2 diabetes mellitus, Jazan area, KSA: A cross-sectional study | Saudi Arabia | 385 | 47.9 (11.4) | M: 47.8%  F: 52.2% | 78.7% | NA | 3.07 (0.89) | 28.05% | NA | 3.08 (1.0) | NA | NA | NA |
| Al Dossari 2019 | Association of vitamin d with glycemic control in Saudi patients with type 2 diabetes: A retrospective chart review study in an emerging university hospital | Saudi Arabia | 200 | 42.4 (14.8) | M:41%  F:59% | NA | NA | NA | 36.5% | 29.0 (6.5) | 7.1 (1.98) | 7.9 (3.7) | 42.5% | NA |
| AL-Eitan 2016 | Evaluation of Glycemic Control, Lifestyle and Clinical Characteristics in Patients with Type 2 Diabetes Treated at King Abdullah University Hospital in Jordan | Jordan | 237 | 56.85 (9.31) | M: 38%  F: 62% | 100% | 40% | 9.20 (8.10) | 60.8% | 32.4 (6.04) | 7.99 (2.20) | 152.82 (73.4) | 66.7% | 9.7% |
| Al Hayek 2014 | Factors Associated with Health-Related Quality of Life among Saudi Patients with Type 2 Diabetes Mellitus: A Cross-Sectional Survey | Saudi Arabia | 283 | 56.4 (13.2) | M:62.9%  F:37.1% | 100% | 26.9% | (<5 - >15) years | NA | NA | NA | NA | NA | NA |
| Ali 2013 | Evaluation of factors associated with inadequate glycemic control and some other health care indicators among patients with type 2 diabetes in Ramallah, Palestine | UAE | 450 | NA | M: 225  F:225 | NA | NA | NA | 70.9% | NA | 7.6 (1.3) | NA | 90.7% | NA |
| Al-Lawati 2012 | HbA1c Levels among Primary Healthcare Patients with Type 2 Diabetes Mellitus in Oman | Oman | 1266 | 53.3 (11.5) | M:45%  F:55% | NA | 12.7% | <5: 54% | 68% | 30.1 (5.7) | 8.2 (2.0) | NA | NA | 6% |
|  |  |  |  |  |  |  |  | >5: 46% |  |  |  |  |  |  |
| Almetwazi 2018 | Factors associated with glycemic control in type 2 diabetic patients in Saudi Arabia | Saudi Arabia | 728 | 45-60 | M:35.16%  F: 64.84% | NA | NA | NA | NA | NA | NA | NA | NA | NA |
| Al-Mukhtar 2012 | General and Gender Characteristics of Type 2 Diabetes Mellitus Among the Younger and Older Age Groups | Iraq | 462 | 52.5 (6.4) | M: 52.8  F: 47.2 | NA | NA | <5:>10 | 78.3% | NA | NA | NA | 34.8% | 51.9% |
| Almutairi 2013 | Direct Medical Cost and Glycemic Control in Type 2 Diabetic Saudi Patients | Saudi Arabia | 300 | 58.3 (13.5) | M: 56%  F: 44% | 72% | NA | NA | 66.67% | NA | NA | NA | NA | NA |
| Al Qahtani 2015 | Impact of family history in glycemic control among type-2 diabetes mellitus patients in Aseer diabetic center | Saudi Arabia | 343 | 59.8 (10.39) | M: 62%  F: 38% | NA | NA | 15.95 (7.55) | NA | 30.37(5.61) | 9.78 (2.12) | NA | NA | NA |
| AL Qudah 2018 | Factors Associated with Poor Hemoglobin A1c Control in Patients with Type 2 Diabetes | Jordan | 171 | 64 (9.8) | M:56.7%  F: 43.3% | 25.7% | 77.2% | 9.9 (7.5) | NA | NA | NA | NA | NA | 49.7% |
| Alramadan 2018 | Glycaemic control for people with type 2 diabetes in Saudi Arabia – an urgent need for a review of management plan | Saudi Arabia | 1111 | 57.6 (11.1) | M: 65.2%  F: 34.8% | 97.9% | NA | 13.9 (8.4) | 54.2% | 69.4 (15.5) | NA | NA | 61.1% | 5.67% |
| Al-Rasheedi 2014 | The Role of Educational Level in Glycemic Control among Patients with Type II Diabetes Mellitus | Saudi Arabia | 384 | 56 (10.91) | M:47.9%  F: 52.1% | 82.3 | 28.7% | <7: >15 | 67.7% | 44 | NA | NA | 50.5 | NA |
| Alromaihi 2019 | Factors Affecting Glycemic Control among Patients with Type 2 Diabetes in Bahrain | Bahrain | 205 | 54.8 (11.7) | M:47.9%  F:52.1% | 33.5% | 63.4% | <5:>10 | 71.1% | NA | NA | NA | 67.6% | 13.9% |
| Al-Shahrani 2012 | Effects of diabetes education program on metabolic control among Saudi type 2 diabetic patients | Saudi Arabia | 438 | 55.84 (10) | M: 63.9%  F: 36.1% | NA | NA | NA | NA | NA | 8.78 (1.78) | 9.99 (2.62) | NA | NA |
| Alzaheb 2018 | The prevalence and determinants of poor glycemic control among adults with type 2 diabetes mellitus in Saudi Arabia | Saudi Arabia | 423 | 30–49 | M:52.7%  F: 47.3% | 93.4% | 43.97% | <5:>10 | 74.9% | NA | NA | NA | 21.5% | 19.38% |
| Alzahrani 2019 | Association between glycated hemoglobin (HbA1c) and the lipid profile in patients with type 2 diabetes mellitus at a tertiary care hospital: a retrospective study | Saudi Arabia | 206 | 60.46 (13.54) | M: 31.5%  F: 68.4% | NA | NA | NA | 56.3% | 30.8 (6.1) | 7.65 (1.78) | 147.7 (58.7) | NA | NA |
| Azadi 2020 | The Relationship of Self-efficacy and Demographic Characteristics With Blood Glucose Control in Iranian Older Adults With Diabetes Type II: A Cross-Sectional Study | Iran | 290 | 71.80 (5.39) | M: 46.2  F: 53.8% | 100% | 14.5% | NA | 51.37% | NA | 7.82 (9.7) | NA | NA | 35% |
| Baltaci 2012 | Effectiveness for self-monitoring of blood sugar on blood glucose control in Turkish patients with type 2 diabetes mellitus | Turkey | 349 | 54.2 (9.7) | M:41.3%  F: 58.7% | 87.1% | 0% | 6.5 (4.9) | 39.2% | 31.5 (5.6) | 7.6 (1.7) | 158.8 (59.5) | NA | 16.9% |
| Baltaci 2015 | Evaluation of Clinical Effectiveness of Self-Monitoring Blood Glucose Level in Patients with Type 2 Diabetes Mellitus Treated with Non Insulin Regimens in Düzce: Primary Care-Based Study | Turkey | 680 | 55.4 (10.8) | M:40.8%  F: 59.2% | 90.2% | NA | 6.9 (4.3) | NA | 32.7 (5.9) | 7.6 (1.6) | NA | NA | 18.8% |
| Channanath 2018 | Glycaemic control in native Kuwaiti Arab patients with type 2 diabetes | Kuwait | 7657 | 50.3 (12.1) | M: 43.9%  F: 56.01% | NA | 26% | 8 (7.35) | 74.4% | 31.8 (6.2) | NA | NA | 36.04% | NA |
| Cosansu 2014 | Influence of Psychosocial Factors on Self-Care Behaviors and Glycemic Control in Turkish Patients With Type 2 Diabetes Mellitus | Turkey | 350 | 53.42 (8.02) | M:40.3%  F: 59.7% | 100% | 41.6% | 8.61 (6.03) | NA | 30.8 (5.35) | 7.34 (1.62) | NA | NA | NA |
| Greenberger 2014 | The inter-relationships between self-efficacy, self-management, depression and glycaemic control in Israeli people with type 2 diabetes | Israel | 600 | 70 years | NA | NA | NA | NA | NA | NA | NA | NA | NA | NA |
| Gucuk 2015 | Effects of the behavior of elderly type 2 diabetic patients and their relatives as caregivers on diabetes follow-up parameters in Bolu, Turkey | Turkey | 115 | 71 (5.01) | M:44.3%  F: 55.7% | 86.1% | NA | 12.14 (6.74) | 44.34% | 30.2 (4.2) | 7.09 (0.64) | NA | 50.4% | NA |
| Habib 2013 | Gender differences in lipid and glycemic control in Saudi patients with Type 2 diabetes mellitus | Saudi Arabia | 1000 | 54.5 (11.23) | M:50.1  F:49.9 | NA | NA | NA | 77.58% | 30.1 (5.39) | 9.36 (3.06) | 10.13 (5.28) | NA | NA |
| Jahanlou 2011 | The Effect of Literacy Level on Health Related-Quality of Life, Self Efficacy and Self-Management Behaviors in Diabetic Patients | Iran | 256 | 49.15 (9.5) | M:32.5%  F: 67.5% | NA | 0% | 6.33 (5.12) | 70% | NA | NA | NA | 16.5% | NA |
| Khattab 2010 | Factors associated with poor glycemic control among patients with Type 2 diabetes | Jordan | 917 | 57.4 (9.6) | M: 455  F: 462 | 88.98% | 37.7% | 9.03 (7.04) | 65.1% | 31.8 (5.7) | NA | NA | 58% | NA |
| Maddah 2016 | Glycemic control and its predictors among Iranian diabetic patients | Iran | 367 | 53.2 (11.3) | M: 35.9%  F: 64.1% | 100% | 15.45% | NA | 72.9% | NA | 7.7 (1.05) | 183.9 (62.5) | NA | NA |
| Mansour 2020 | Prevalence and correlation of glycemic control achievement in patients with type 2 diabetes in Iraq: A retrospective analysis of a tertiary care database over a 9-year period | Iraq | 12869 | 51.4 (11.34) | M:45.6%  F: 54.4% | NA | 54.2% | 9.7 (11.34) | 75.7% | 29.9 (11.3) | 10.1 (22.68) | NA | NA | NA |
| Megahed 2020 | Most Effiecnt Factors Affecting Glycemic Control of Type II Diabetic Patients Attending Suez Canal University Hospitals in Egypt Applying Stepwise Regression | Egypt | 92 | 49.76 (9.19) | M: 31.5%  F: 68.5% | 57.6% | NA | 2.63 (1.6) | 65.2% | 32.5 (5.65) | NA | NA | 60.9% | 3.3% |
| Mirahmadizadeh 2019 | Adherence to Medication, Diet and Physical Activity and the Associated Factors Amongst Patients with Type 2 Diabetes | Iran | 500 | 56.92 (0.52) | M: 41.2%  F:58.8% | 79.6% | 28.4% | NA | 60.2% | NA | 7.48 (0.06) | NA | 22.1% | 12.8% |
| Mosleh 2017 | Predictors of Good Glycemic Control Among Type Ii Diabetes Patients In Palestine | Palestinian | 330 | 60 (9.7) | M:51.2%  F: 48.8% | 89.7% | 76.4% | 14.0 (9‑20) | 79.7% | NA | 8.4 (1.7) | NA | 47.2% | 36.2% |
| Nemeh 2011 | Glycemic Control and Its Determinants among Patients with type 2 Diabetes Mellitus Attending a Teaching Hospital | Jordan | 533 | 50.1 (14.1) | M: 38%  F: 62% | NA | NA | NA | 40.3% | 27.6 (3.5) | NA | NA | 22.3% | 75.9% |
| Noureddine 2014 | Level of A1C control and its predictors among Lebanese type 2 diabetic patients | Lebanon | 551 | 58.5 (12.4) | M: 57.5%  F: 42.4% | NA | 32.3% | 8.2 (7.5) | 68.2% | 30.5 (5.5) | NA | NA | 46% | 56.1% |
| Nozha 2014 | Diabetes care and control: the effect of frequent visits to diabetes care center | Saudi Arabia | 100 | 52.0 (15.2) | M: 42%  F: 58% | 90% | 17% | 12 (2.1) | NA | 28.8 (6.4) | 8.3 (1.6) | NA | NA | 32% |
| Qteishat 2015 | Comprehensive assessment of variables affecting metabolic control in patients with type 2 diabetes mellitus in Jordan | Jordan | 200 | 53.53 (10.38) | M:40%  F: 60% | 83% | 27.5% | NA | 67% | NA | 8.4 (1.95) | NA | 55% | NA |
| Radwan 2017 | Glycemic control among primary care patients with type 2 diabetes mellitus in the Gaza Strip, Palestine | Palestine | 369 | 56.38 (10.36) | M: 44.1%  F: 55.8% | NA | 23% | 10.48 (8.1) | 80.4% | NA | 8.97 (2.02) | NA | NA | NA |
| Saad 2017 | Self-efficacy, Self-care and Glycemic control in Saudi Arabian patients with type 2 Diabetes Mellitus: A Cross-sectional Survey | Saudi Arabia | 123 | 61.97 (11.53) | M: 72.4%  F: 27.6% | 73.2% | 47.1% | NA | NA | NA | 7.6 (1.7) | NA | 43.1% | 13% |
| Saghir 2019 | Factors associated with poor glycemic control among type-2 diabetes mellitus patients in Yemen | Yemen | 246 | 49.5 (11.8) | M: 52.4%  F: 47.6% | 70.7% | 25.2% | 7.1 (5.1) | 73.2% | 25.8 (4.9) | 8.8 (2.2) | NA | 16.7% | 29.3% |
| Samancioglu 2017 | Factors Affecting Glycemic Control in Type 2 Diabetics and Diabetics’ Attitude towards the Disease | Turkey | 341 | NA | M: 36.1%  F: 63.9% | 100% | 62.8% | NA | 22.9% | NA | 9.80 (3.43) | NA | NA | NA |
| Samara 2017 | Prevalence of Glycemic Control and Factors Associated With Increasing Levels of Hba1c Among A Sample of Palestinian Patients With Type 2 Diabetes Mellitus | Palestine | 380 | 11.2 (8.2) | M: 42.1%  F: 57.9% | 86.8% | 58.4% | 11.2 (8.2) | 73.2% | 32.7 (6) | 8.3 (1.8) | NA | 68.9% | 26.3% |
| Sürücü 2017 | Empowerment and Social Support as Predictors of Self-Care Behaviors and Glycemic Control in Individuals With Type 2 Diabetes | Turkey | 220 | 53.63 (11.48) | M: 46.4%  F: 53.6% | 73.1% | 74.5% | 10.17 (7.95) | NA | NA | 9.95 (2.22) | NA | NA | NA |
| Tol 2011 | The Relationship between perceived social support from family and diabetes control among patients with diabetes type 1 and type 2 | Iran | 317 | 35-60 | M: 45.4%  F: 54.6% | 68.1% | NA | NA | 45.4% | NA | NA | NA | 23.7% | NA |
| Yacoub 2011 | Effect of Frequent Self-Monitoring of Blood Glucose on HbAlc Level Among Type 2 Diabetic Patients | Iraq | 126 | 41.58 (13.8) | M:50.7%  F: 49.3% | 100% | 77.78% | NA | NA | 27.05(4.9) | 7.01 (1.23) | NA | NA | NA |

Table 1: Summary of included studies and patients

NA: Not available; M: Male; F: Female
